# Heart Rate Variability: A Psychophysiological Factor Related to Both Regular Physical Activity and Eudaimonic Well‐Being Among Young Adults

**DOI:** 10.1002/brb3.70284

**Published:** 2025-01-26

**Authors:** Yoshino Murakami, Daisuke Goto, Hayato Tsukamoto, Kazuho Yamaura, Takeshi Hashimoto

**Affiliations:** ^1^ Faculty of Sport and Health Science Ritsumeikan University Kusatsu Japan; ^2^ Faculty of Sport Sciences Waseda University Tokorozawa Japan

**Keywords:** electrocardiogram, moderate‐intensity PA, neurotransmitters, psychological well‐being

## Abstract

**Introduction:**

Eudaimonic well‐being (EWB), which refers to optimal human functioning, is associated with psychophysiological outcomes, such as reduced inflammation and a lower risk of depression. Although physical activity (PA) and mindfulness have been shown to be predictive factors for EWB, potential mediators of the relationships of PA or mindfulness with EWB have yet to be identified. PA, mindfulness‐related psychophysiological factors (including serotonin [5‐HT], oxytocin [OXT]), and dopamine [DA] levels), and heart rate variability (HRV) have been shown to be associated with mental disorders or emotion regulation capacity.

**Purpose:**

The purpose of this study is to explore the potential psychophysiological factors associated with PA, mindfulness and EWB.

**Methods:**

A total of 49 young adults (25 males, 24 females) were included with an average age of 25 years (± 5). Plasma 5‐HT, OXT, and DA levels were obtained via blood samples from the brachial vein and were analyzed with enzyme‐linked immunosorbent assays (ELISAs), and HRV was obtained via 5‐min electrocardiograms (ECGs), with participants in the supine position. Spearman's correlation analyses were performed, followed by partial correlation analyses controlling for age, sex, and social status (i.e., student or working professional).

**Results:**

HRV was found to be positively correlated with both moderate‐intensity PA (*r* = 0.47, *p* = 0.04) and EWB (purpose in life; *r* = 0.50, *p* = 0.03), even after controlling for relevant variables. On the other hand, neither 5‐HT, OXT, nor DA was correlated with PA, mindfulness, and EWB.

**Conclusion:**

These results suggest that HRV may mediate the relationship between PA and EWB. Additional intervention studies are needed to elucidate the causal relationships among PA, HRV, and EWB.

## Graphical abstract

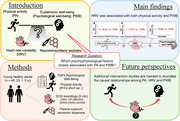

## 1 Introduction

Mental health involves not only the absence of mental illness but also the presence of positive feelings (e.g., hedonic well‐being [HWB]) and positive functioning in individual life (e.g., eudaimonic well‐being [EWB]; Lamers et al. 2011). Previous research on mental health has often focused on psychological conditions such as depression and anxiety while neglecting positive aspects of mental health (i.e., well‐being). Indeed, an increasing number of publications have emphasized the need to shift the focus of mental health research from negative (psychological disorders) to positive (well‐being; Jeste et al. 2015; Seligman and Csikszentmihalyi 2000; Zhang and Chen 2019).

Recent research has divided well‐being into hedonic and eudaimonic concepts, and their associations with psychophysiological biomarkers have been studied (Huppert 2009; Huta and Ryan 2010; Huta and Waterman 2014). HWB refers to the pursuit of pleasure and the presence of life satisfaction, whereas EWB refers to optimal human functioning (Ryff 2013). EWB is evaluated by the Psychological Well‐Being (PWB) scale, which was developed by Ryff (1989). The PWB scale is a multidimensional scale composed of six dimensions: (1) purpose in life, (2) personal growth, (3) positive relations, (4) autonomy, (5) self‐acceptance, and (6) environmental mastery.

Individuals with higher EWB exhibit lower levels of psychophysiological stress indicators, such as cortisol and interleukin‐6 (IL‐6) levels (Lindfors and Lundberg 2002; Ryff, Singer, and Love 2004), whereas HWB has shown limited associations with biological markers (Ryff 2013). Similarly, lower EWB has been identified as a risk factor for depression, as individuals with low EWB have a two‐fold higher risk of developing depression 10 years later (Ruini and Cesetti 2019; Wood and Joseph 2010). Furthermore, EWB has been reported to be more beneficial for job performance than HWB (Peiró, Kozusznik, and Soriano 2019). In particular, young adults face various stresses (academic, social, media influence, etc.; Ruini and Cesetti 2019), resulting in a greater risk of developing mental disorders than older individuals do (Westerhof and Keyes 2010). Hence, improving EWB among young people who are experiencing psychologically unstable periods is a significant issue not only for future disease prevention but also for enhancing their performance in daily life.

### 1.1 Relationships of PA and Mindfulness With PWB

Multiple studies have suggested that physical activity (PA) and mindfulness may be predictive factors for PWB. Saunders, Huta, and Sweet (2018) followed individuals after cardiac rehabilitation and reported that individuals with higher levels of moderate‐vigorous PA (MVPA) at baseline (immediately after rehabilitation) were more likely to have higher EWB 3 months later (*β* = 0.13). Moreover, engagement in leisure‐time PA among lonely older adults was found to positively predict PWB (Kim et al. 2017). Even in individuals experiencing loneliness, engaging in leisure‐time PA may provide psychological benefits. Similarly, higher levels of PA and moderate‐ to high‐intensity PA have been shown to be associated with higher PWB among younger populations (Lapa 2015; Ugwueze et al. 2021). Intervention studies have also provided evidence regarding the effects of PA on PWB. For example, 12 weeks of a moderate‐intensity aerobic exercise program for older adults resulted in significant improvements in PWB (Shams et al. 2021). Furthermore, 8 weeks of a Zumba exercise program for young healthy women led to a meaningful increase in PWB (Delextrat et al. 2016). However, the specific factors that influence the effects of PA on EWB have yet to be identified. Mindfulness is defined as “paying attention in a particular way: On purpose, in the present moment, and nonjudgmentally” (Kabat‐Zinn 1990). The Five‐Facet Mindfulness Questionnaire (FFMQ) is commonly used as a comprehensive measure to assess various aspects of trait mindfulness in daily life (Kabat‐Zinn 1990). In university students, FFMQ scores are positively correlated with PWB and were identified as a direct predictor of EWB (Bowlin and Baer 2012; Jarukasemthawee and Pisitsungkagarn 2021). Furthermore, trait mindfulness has been cultivated through practices such as mindfulness‐based interventions (MBIs; Keng, Smoski, and Robins 2011). Several studies involving various populations, including breast cancer patients and healthy individuals, have demonstrated that MBIs lead to improvements in EWB (Haji‐Seyed‐Sadeghi et al. 2020; Kosugi et al. 2021). Therefore, both PA and mindfulness (trait mindfulness and MBIs) clearly contribute to the improvement of EWB. However, little is known about the underlying physiological factors of these effects. To determine the optimal intervention protocols to promote health, it is important to identify the mediators that underlie the relationships among PA, mindfulness, and PWB.

### 1.2 Relationships Between Neurotransmitters and PWB

Alterations in neurotransmitters and neuropeptides, such as serotonin (5‐HT), oxytocin (OXT), and dopamine (DA), have been identified as factors contributing to mental and behavioral disorders and may lead to a decline in mental health (de Vries, van de Weijer, and Bartels 2022; Walker and McGlone 2013). For example, 5‐HT and DA are associated with a positive mood, whereas OXT plays a role in fostering social bonding and enhancing emotional connections with others (de Vries, van de Weijer, and Bartels 2022; Baixauli 2017; Farhud et al. 2014). In this context, higher levels of these three neurotransmitters may be associated not only with a reduction in depressive symptoms but also with positive psychological aspects, such as well‐being. However, in their systematic review, de Vries et al. summarized findings on the associations between psychologically related physiological factors (psychophysiological factors) and EWB/HWB and reported that most studies have used HWB as a well‐being indicator, whereas very few studies have assessed EWB.

### 1.3 Relationship Between Heart Rate Variability (HRV) and PWB

In addition, HRV, which is mediated by the vagus nerve, has also been examined as a psychophysiological index of emotion regulation capacity, as the vagus nerve is connected to the same neural network (Koval et al. 2013; Williams et al. 2015). A neuroanatomical study revealed that HRV is associated with more adaptive and functional cognitive processing of emotional stimuli, which may facilitate effective emotion regulation (Thayer and Lane 2000, 2009). Furthermore, it has been suggested that emotion‐regulation processes (e.g., goal setting, goal striving, goal achievement, etc.) are important factors for PWB (Harzer 2016; Toh and Yang 2022). In this context, HRV could be linked to higher levels of PWB. Interestingly, only one study assessed the relationship between EWB and HRV, but no relationship was detected; the lack of relationship could be attributed to the time lag of assessing PWB and HRV (Sloan et al. 2017). To better understand how PA and mindfulness contribute to improving EWB, it is important to identify the psychophysiological factors that potentially mediate these relationships.

As mentioned above, activities such as PA and MBIs, which promote EWB, have been suggested to regulate psychophysiological factors, including those of the immune system, autonomic nervous system (ANS) and neuroendocrine system, leading to improved mental health (e.g., preventing mood disturbance). For example, both PA and MBIs have been reported to reduce subjective stress and the levels of inflammatory‐related biomarkers (e.g., cortisol and IL‐6) under basal conditions, mostly in older adults and patients with chronic conditions (Creswell and Lindsay 2014; Melanson 2000; Paolucci et al. 2018; Saban et al. 2022). They have also been associated with improved ANS function, indicated by an increase in parasympathetic nervous system (PNS) activity as observed with HRV. High HRV is associated with improved regulatory and homeostatic ANS functions, which increase the body's ability to cope with internal and external stressors (Kim et al. 2018); hence, high HRV might be beneficial for mental health. Furthermore, acute and/or long‐term exercise has been found to increase the levels of neurotransmitters/neuropeptides (5‐HT, DA, and OXT; Albantakis et al. 2021; Meeusen and Piacentini. 2001), and long‐term exercise enhances PNS activity (Herbert et al. 2020), which could contribute to mood enhancement.

### 1.4 Purpose and Hypotheses

The aim of this study was to explore the potential psychophysiological factors associated with PA, mindfulness, and EWB. Identifying the psychophysiological factors closely associated with PA or mindfulness and EWB could provide essential insights into the mediators underlying the effects of PA and mindfulness on EWB improvement. Because lower levels of psychophysiological factors have been shown to be associated with various psychological issues (e.g., difficulties in emotion regulation), we hypothesized that psychophysiological factors would be positively correlated with PA, mindfulness and EWB.

## 2 Methods

### 2.1 Ethics Approval

The study was approved by the Institutional Review Board (IRB) of Ritsumeikan University (IRB approval number: BKC‐LSMH‐2021‐056‐2).

### 2.2 Participants

Healthy young individuals (mean ± SD: age = 25 ± 5 years, height = 166.8 ± 8.2 cm, body mass = 59.6 ± 10.9 kg; sex ratio: 51% male, 49% female) were recruited via a combination of purposive, snowball, and digital‐based sampling methods. The recruitment process involved poster advertisements displayed in university facilities and the distribution of recruitment flyers through messaging applications and email, thus allowing participants to further share the materials within their personal networks and on social media.

### 2.3 Inclusion and Exclusion Criteria

The inclusion criteria for this study were (1) healthy young adults aged between 20 and 39 years and (2) individuals with an interest in participating in the study. The exclusion criteria were as follows: (1) individuals currently diagnosed by a physician with any of the following conditions: addiction, depression, anxiety disorder, eating disorder, bipolar disorder, epilepsy, schizophrenia, or dementia; (2) regular use of prescribed medications or psychotropic drugs for mental health improvement; (3) regular visits to a physician for mental and/or physiological issues; or (4) females with irregular or absent menstruation.

### 2.4 Procedures

The participants visited the laboratory at Ritsumeikan University, Biwako‐Kusatsu, in the morning (at 8:00–11:30 a.m.) for a single day between June and September 2022. The participants were informed about the study and provided informed consent before their participation. Data collection involved a questionnaire (demographic characteristics, PWB, PA, mindfulness) and physiological measurements (height, weight, body mass index [BMI], electrocardiogram [ECG] recording and blood sampling). All the physiological measurements were completed before 12:00 p.m. The participants were instructed to breathe spontaneously during the ECG recordings to capture their inherent traits. We targeted female participants with stable menstrual cycles, confirmed their menstrual start date in the past 3 months, and scheduled the experiments during the premenstrual phase (defined as the luteal phase). To control for the effects of other lifestyle factors on the results, participants were instructed to refrain from alcohol consumption, intense exercise and caffeine intake starting on the day before the experiment. They were also instructed to get sufficient sleep, have breakfast at least 3 h before the experiment, and avoid excessive PA when commuting to the laboratory (e.g., use the bus or other forms of transportation). The laboratory environment was carefully maintained at constant conditions (room temperature [°C:] 23.4 ± 2.6, humidity [%]: 64 ± 7).

### 2.5 Questionnaires

#### 2.5.1 EWB

To evaluate EWB, the PWB scale, which was originally developed by Ryff (1989), and the 43 item Japanese version translated by Nishida (2000) were used in this study. The PWB scale is a self‐rating measurement that covers six dimensions of well‐being: purpose in life (eight items), personal growth (eight items), positive relations (seven items), autonomy (seven items), self‐acceptance (seven items), and environmental mastery (six items). The items are rated on a scale from 1 (*strongly disagree*) to 6 (*strongly agree*). Thus, the score of each subscale ranges from 1 to 6. Total scores on the PWB scale are calculated by summing the average score in each dimension and ranges from 6 to 36. A higher score represents a higher level of well‐being. The internal consistency (*α*) values ranged from 0.74 (environment mastery) to 0.89 (purpose in life).

#### 2.5.2 PA

To assess PA, the self‐reported, short version of the International Physical Activity Questionnaire (IPAQ) was used (Murase et al. 2002). The participants were asked about the frequency and duration of high‐intensity PA, moderate‐intensity PA, walking activity, and sedentary time in the previous week. Calculations were conducted for the following metrics: ① high‐intensity PA [metabolic equivalents (METs) × min/week], ② moderate‐intensity PA [METs × min/week], ③ walking activity [METs × min/week], ④ sedentary time [min/week], and ⑤ total PA [METs × min/week] (①+②+③). MET is defined as the metabolic equivalent and is a unit of intensity of activity. 1‐MET is equivalent to the intensity of resting while sitting. The MET values used in the calculation included 8 METs for high‐intensity PA, 4 METs for moderate‐intensity PA and 3.3 METs for walking activity. The reliability and validity of this questionnaire have been reported in 12 countries, including Japan. The test–retest reliability for total PA of the Japanese IPAQ was adequate (Spearman's rho = 0.76). The criterion validity for total PA assessed against the accelerometer was comparable to that of other survey measures (Spearman's rho = 0.32; Craig et al. 2003).

#### 2.5.3 Trait Mindfulness

The FFMQ (Sugiura et al. 2012) was used to measure trait mindfulness. The FFMQ is a 39‐item self‐report questionnaire that consists of the following five domains of mindfulness awareness: (1) observing, (2) describing, (3) acting with awareness, (4) nonjudging of internal experience, and (5) nonreactivity to inner experience. The questionnaire includes the following items: “When I'm walking, I deliberately notice the sensations of my body moving” and “I perceive my feelings and emotions without having to react them” (Bohlmeijer et al. 2011). The items are rated on a 5‐point Likert scale ranging from 1 (*never or very rarely true*) to 5 (*very often or always true*). The FFMQ total score is calculated as the sum of the average score of each dimension and ranges from 5 to 25. Higher scores indicate greater dispositional mindfulness. The internal consistency (*α*) values of the FFMQ ranged from 0.68 (observation) to 0.84 (nonjudging) in the current sample.

### 2.6 Resting HRV

ECG (sampling frequency = 128 Hz) data were recorded for 10 min while the participants were at rest in the supine position via the myBeat WHS‐1 (Union tool. Co, Tokyo, Japan). To measure the ECG, myBeat WHS‐1 and electrodes were attached directly to the participant's chest by a dedicated chest strap (Union tool. Co, Tokyo, Japan). Artifacts in the ECG data were automatically corrected following a visual inspection of the ECG signal (percentage of corrected beats: 1.40% ± 2.13%). After artifact correction, R–R interval series were derived from the ECG data via LabChart v7. The R‒R intervals were interpolated with an 8‐Hz resampling rate. HRV was analyzed in both the time domain (root mean square of successive differences [RMSSD], percentage of successive R‒R interval series that differed by more than 50 ms [pNN50 (%)], number of successive NN intervals that differed by more than 50 ms [NN50]) and frequency domain (high‐frequency [HF]: 0.15–0.4 Hz), which are considered to reflect vagal tone and/or are closely correlated with PNS activity (Task force of the European Society of Cardiology and the North American Society of Pacing and Electrophysiology, 1996; Shaffer, McCraty, and Zerr 2014), during the final 5 min of recording. The power spectral density of the interpolated R‒R intervals was computed via fast Fourier transform (FFT) with Welch's periodogram method. All HRV indices were calculated with normalized units via the open‐source Python package hrv (version 0.2.8, available at https://hrv.readthedocs.io/en/latest/index.html#; Bartels and Peçanha 2020).

### 2.7 Blood Sampling

Following ECG recording, participants maintained a supine position, and venous blood (5 mL) was collected via a 22‐gauge needle (0.70 × 16 mm) into the EDTA tube from the median cubital vein in the nondominant forearm unless sampling from the arm, in which the dominant forearm was targeted, was difficult. After collection, the blood samples were immediately cooled on ice and subjected to centrifugation (4°C, 3000 rpm, 10 min). The plasma was then extracted and stored at −80°C until quantification. Enzyme‐linked immunosorbent assay (ELISA) kits were used to measure 5‐HT (BA‐E‐5900R, Immusmol, France), OXT (EKE‐051‐01, Phoenix Pharmaceuticals, USA) and DA levels (KA‐1887, Abnova, Taiwan). The detection limits were 0.015 ng/mL for 5‐HT, 0.08 ng/mL for OXT, and 3.3 pg/mL for DA, and the intra‐assay coefficients of variation (CVs) were estimated to be 2.63% for 5‐HT, 3.87% for OXT, and 1.36% for DA.

### 2.8 Statistical Analysis

After the Shapiro‒Wilk test was performed to assess the normality of the variables, Spearman's correlation analysis was conducted to examine the relationships of psychophysiological factors with PWB scores, IPAQ scores, and FFMQ scores. Subsequently, partial correlation analysis was performed, controlling for age, sex and social status (students or working professionals). All the statistical analyses were conducted via IBM SPSS software (Version 29.0, IBM Corp., Armonk, NY, USA), and the significance threshold was set at *p* < 0.05.

## 3 Results

A total of 49 participants were enrolled in this study. The demographic characteristics of the participants are presented in Table 1. The mean age ± SD was 25 ± 5 years. Both sex and social status were balanced, with nearly equal representation (males: *n* = 25 [51%]; students: n = 25 [51%]). The participants’ mean BMI was within the normal range (21.3 ± 2.8 kg/m^2^), which is consistent with the World Health Organization's (WHO) classification for normal weight (18.5–24.9 kg/m^2^; WHO 2021). No medication endorsements were reported. With respect to the ELISA data, only detectable data were included (5‐HT: *n* = 49, OXT: *n* = 48, DA: *n* = 43). Some ECG data were not recorded properly because of connectivity issues; these data were excluded, and thus 26 samples were included for HRV analysis (Table 2).

**TABLE 1:** Descriptive statistics of demographic characteristics.

Demographic variables	Mean	(SD)	Min	Max
Mean age (SD)	25	(5)	20	38
Mean body mass index (SD)	21.3	(2.8)	17.7	37.3
Sex	*n*	(%)		
Male	25	(51.0)		
Female	24	(49.0)		
Race				
Asian	49	(100.0)		
Other	0	(0.0)		
Social status				
Student	25	(51.0)		
Working professional	24	(49.0)		
Marital status				
Yes	7	(14.3)		
No	42	(85.7)		
Alchohol				
Yes	16	(32.7)		
No	33	(67.3)		
Smoking				
Yes	2	(4.1)		
No	47	(95.9)		

*Note: n* = 49.

**TABLE 2:** Scores on the key variables.

Variables	*N*	Mean	Median (Q1, Q3)	Range	
Psychological Well‐Being (PWB)					
Purpose in life	49	4.30	4.25 (3.63, 5.00)	1–6	
Personal growth	49	5.40	5.63 (4.88, 5.94)	1–6	
Positive relations	49	4.62	4.83 (4.09, 5.25)	1–6	
Autonomy	49	3.93	4.00 (3.38, 4.63)	1–6	
Self‐acceptance	49	3.75	3.71 (3.07, 4.36)	1–6	
Environmental mastery	49	4.40	4.17 (4.00, 4.75)	1–6	
PWB total	49	26.40	25.77 (24.36, 28. 43)	6–36	
Mindfulness (FFMQ)		Mean	Median (Q1, Q3)	Range	
Observation	49	2.93	2.75 (2.50, 3.50)	1–5	
Non‐reactivity	49	2.98	3.00 (2.57, 3.43)	1–5	
Non‐judging	49	3.22	3.25 (2.63, 3.63)	1–5	
Description	49	3.07	3.00 (2.50, 3.56)	1–5	
Acting with awareness	49	3.27	3.13 (2.75, 3.75)	1–5	
FFMQ total	49	15.49	15.39 (14.30, 16.25)	5–25	
Physical activity (PA; IPAQ)		Mean	Median (Q1, Q3)	Min	Max
Sedentary time (min/week)	49	430	360 (180, 600)	30	1080
Walking activity (METs·min/week)	49	633	594 (198, 891)	0	2079
Moderate intensity PA (METs·min/week)	49	480	240 (0, 480)	0	3240
High‐intensity PA (METs·min/week)	49	669	0 (0, 480)	0	6000
Total PA (METs·min/week)	49	1795	1152 (570, 2564)	0	8079
Psychophysiological factors		Mean	Median (Q1, Q3)	Min	Max
Neurotransmitters/neuropeptides					
5‐HT (ng/mL)	49	22.30	17.96 (11.34, 31.30)	2.90	59.40
Oxytocin (ng/mL)	48	2.84	2.38 (2.02, 3.16)	1.64	8.68
Dopamine (pg/mL)	43	40.48	34.77 (16.15, 60.76)	0.20	123.15
Heart rate variability (HRV) indexes					
RMSSD (ms)	26	72.02	53.58 (64.87, 114.14)	22.32	186.29
SDNN (ms)	26	71.20	80.74 (67.92, 107.86)	48.05	160.61
pNN50 (%)	26	33.35	27.07 (11.28, 56.40)	3.14	77.53
NN50	26	102.12	99.00 (38.00, 156.50)	12.00	210.00
HF (nu)	26	64.72	66.55 (53.18, 77.56)	29.78	89.77

*Note*: FFMQ: Five‐Facet Mindfulness Questionnaire. IPAQ: International Physical Activity Questionnaire. “Range” refers to the minimum to maximum scores on each scale.

### 3.1 Normality Test

The Shapiro–Wilk test indicated that most variables significantly deviated from normality (*p* < 0.05), including sedentary time, walking activity, moderate PA, high‐intensity PA, total PA, 5‐HT, OXT, DA, personal growth, environment mastery, RMSSD, SDNN, and pNN50. On the other hand, the following variables followed a normal distribution (*p* > 0.05): mindfulness factors such as nonreactivity (*p* = 0.93), nonjudgment (*p* = 0.64), acting with awareness (*p* = 0.56); PWB factors such as purpose in life (*p* = 0.76), autonomy (*p* = 0.56), and self‐acceptance (*p* = 0.54); and HF (*p* = 0.31).

### 3.2 PA, Mindfulness, and PWB

**FIGURE 1:**
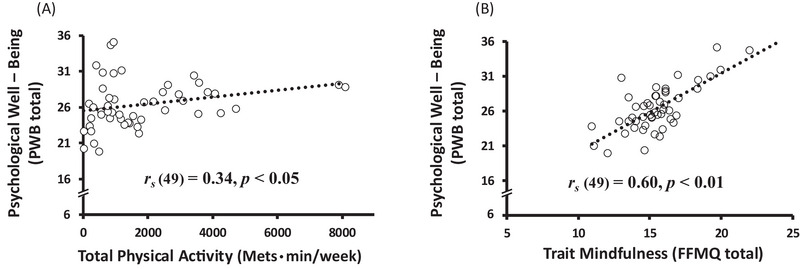
Correlations of PA and mindfulness with PWB. *Note*: (A) The relationship between PA and PWB scores. (B) The relationship between mindfulness (FFMQ scores) and PWB scores. FFMQ: Five‐Facet Mindfulness Questionnaire; PA: physical activity; PWB: Psychological Well‐Being Scale.

Consistent with previous reports, both PA (total PA [METs × min/week]) and mindfulness (FFMQ scores) were positively correlated with total scores on the PWB scale (*r_s_* = 0.34, *p* < 0.05 and *r_s_* = 0.60, *p* < 0.01; Figure 1).

### 3.3 PA, Mindfulness, and Psychophysiological Factors

**FIGURE 2:**
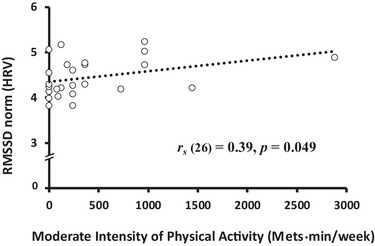
Correlation between moderate‐intensity PA and RMSSD (HRV). *Note*: The relationship between PA and the time domain of the HRV index (RMSSD).

**FIGURE 3:**
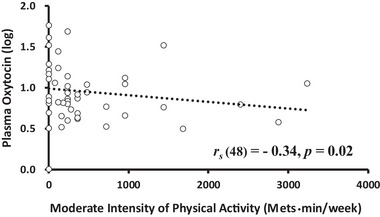
Correlation between moderate‐intensity PA and OXT levels. *Note*: Plasma OXT levels were natural logarithm (log) transformed due to the high variability among individuals; the absolute value was used for the analysis.

**FIGURE 4:**
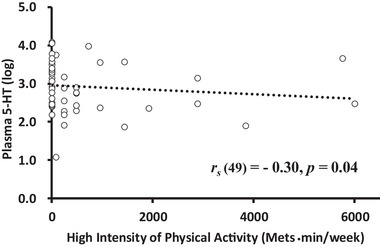
The relationship between high‐intensity PA and 5‐HT levels. *Note*.Plasma5‐HT levels were natural logarithm (log) transformed due to the high variability among individuals; the absolute value was used for the analysis.

**FIGURE 5:**
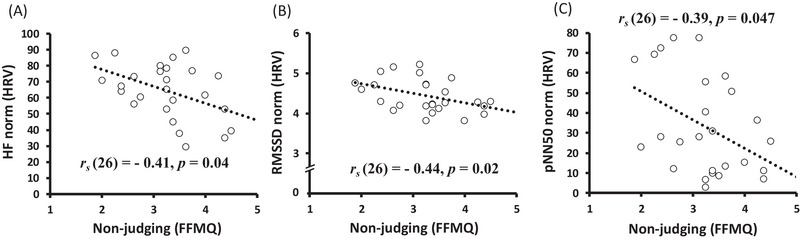
Correlations between mindfulness and HRV indices. *Note*: The relationship between nonjudging (mindfulness) and HRV indices. Nonjudging involves observing one's inner experience without evaluation or judgment.

The data in Tables 3 and 4 show the correlations among the IPAQ, FFMQ, and psychophysiological factors. The coefficients that were statistically significant are highlighted in bold, and the scatter plots of these variables are provided below the tables (Figures 2, 3, 4, 5).

**TABLE 3:** The correlations between PA and psychophysiological factors.

PA	Neurotransmitters/neuropeptides			HRV indices				
	5‐HT (ng/mL)	Oxytocin (ng/mL)	Dopamine (pg/mL)	RMSSD (nu)	SDNN (nu)	NN50 (nu)	pNN50 (nu)	HF (nu)
Sedentary time (min/week)	0.15	−0.23	0.01	−0.06	0.05	−0.20	−0.13	0.07
Walking activity (METs·min/week)	−0.03	0.01	0.06	−0.20	−0.20	−0.16	−0.15	0.16
Moderate PA (METs·min/week)	−0.11	−0.34^*^	−0.07	0.39^*^	0.33	0.34	0.32	0.29
High PA (METs·min/week)	−0.30^*^	0.06	0.03	0.30	0.26	0.26	0.30	0.19
Total PA (METs·min/week)	−0.17	−0.03	0.19	0.22	0.17	0.13	0.16	0.37

*Note*: Moderate PA: moderate‐intensity physical activity. High PA: high‐intensity physical activity. HRV indices include HF: high frequency [0.15−0.40 Hz], RMSSD, SDNN, NN50 and pNN50 (%), and all these variables are shown in normalized units (nu). The coefficients that were statistically significant are highlighted in bold.

**TABLE 4:** The correlation coefficients of the relationships between mindfulness and psychophysiological factors.

Mindfulness	Neurotransmitters/neuropeptides			HRV indices				
	**5‐HT (ng/mL)**	**Oxytocin (ng/mL)**	**Dopamine (pg/mL)**	**RMSSD (nu)**	**SDNN (nu)**	**NN50 (nu)**	**pNN50 (nu)**	**HF (nu)**
FFMQ total	−0.02	−0.07	0.14	−0.28	−0.22	−0.11	−0.18	−0.13
Observation	−0.07	−0.15	0.04	−0.10	−0.14	0.22	0.13	0.32
Non‐reactivity	−0.08	0.06	0.06	−0.20	−0.16	−0.16	−0.23	−0.20
Non‐judging	−0.05	−0.05	0.15	−0.44^*^	−0.34	−0.37	−0.39^*^	−0.41^*^
Description	0.02	0.08	0.04	−0.26	−0.27	0.01	−0.09	0.06
Acting with awareness	−0.08	−0.24	0.07	0.06	0.12	0.11	0.08	0.10

*Note*. Significance level: ^*^ *p* < 0.05.

The coefficients that were statistically significant are highlighted in bold.

Moderate‐intensity PA was positively correlated with the RMSSD (HRV; *r_s_* = 0.39, *p* = 0.049; Figure 2) and negatively correlated with plasma OXT levels (ng/mL; *r_s_* = −0.34, *p* = 0.02; Figure 3). Additionally, high‐intensity PA was negatively correlated with plasma 5‐HT levels (ng/mL; *r_s_* = −0.30, *p* = 0.04; Figure 4). Non‐judging dimention of mindfulness was negatively correlated with HRV (vs. HF; *r_s_* = ‐ 0.41, *p* < 0.05, vs. RMSSD; *r_s_* = ‐ 0.44, *p* < 0.05, vs. pNN50; *r_s_* = ‐ 0.39, *p* < 0.05) (Figure 5).

### 3.4 Psychophysiological Factors and PWB

**FIGURE 6:**
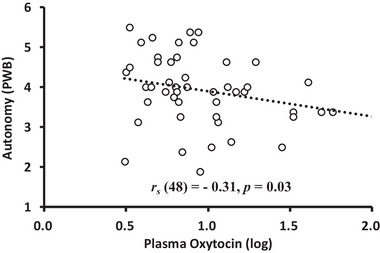
The relationship between plasma OXT levels and autonomy (PWB scores). *Note*: Plasma OXT levels were natural logarithm (log) transformed due to the high variability among individuals; the absolute value was used for the analysis.

**FIGURE 7:**
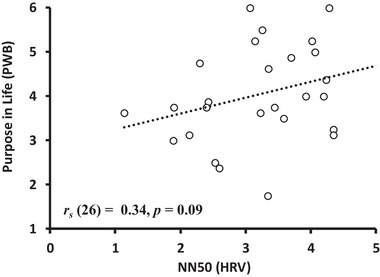
The relationship between NN50 (HRV) and purpose in life (PWB scores). *Note*: Correlation between the time domain of the HRV index (NN50) and PWB scores.

**FIGURE 8:**
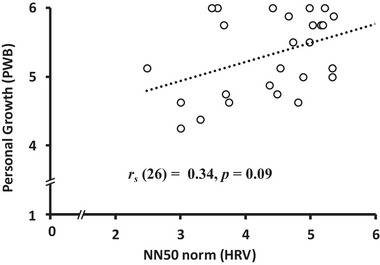
The relationship between NN50 (HRV) and personal growth (PWB scores) *Note*: Correlation between the time domain of the HRV index (NN50) and PWB scores.

OXT levels were negatively correlated with scores on the autonomy dimension of the PWB scale (*r_s_* = −0.31 *p* = 0.03; Figure 6). Additionally, there was a marginally significant positive correlation between NN50 (HRV) and the purpose in life and personal growth dimensions of the PWB scale (*r_s_* = 0.34, *p* = 0.09 and *r_s_* = 0.34, *p* = 0.09, respectively; Figures 7 and 8). There were no other significant relationships or trends between the variables (Table 5).

**TABLE 5:** The correlation coefficients of the relationships between psychophysiological factors and PWB scores.

Psychophysiological factors							
	PWB total	Purpose in life	Personal growth	Positive relations	Autonomy	Self‐acceptance	Environmental mastery
Neurotransmitters/neuropeptides							
5‐HT (ng/mL)	−0.23	−0.20	−0.18	−0.03	−0.21	−0.06	−0.08
Oxytocin [OXT] (ng/mL)	−0.08	−0.12	−0.10	0.03	−0.31^*^	0.00	−0.04
Dopamine [DPA] (pg/mL)	0.13	0.08	0.24	0.14	−0.08	0.22	0.08
HRV							
RMSSD (nu)	0.17	0.23	0.28	0.04	−0.07	−0.03	−0.11
SDNN (nu)	0.16	0.23	0.29	0.00	−0.08	−0.01	−0.07
NN50 (nu)	0.24	0.34^#^	0.34^#^	0.14	0.05	0.08	0.11
pNN50 (nu)	0.21	0.27	0.31	0.15	−0.05	0.05	0.05
HF (nu)	0.23	0.29	0.19	0.19	0.13	−0.15	0.13

*Note*: Significance level: ^#^ *p* < 0.1, ^*^ *p* < 0.05. The coefficients that were statistically significant are highlighted in bold.

### 3.5 Partial Correlations

Age, sex and social status (student or working professional) have frequently been reported to be related to PA, HRV, and PWB. Table 6 shows the correlations between these cofounding factors and PA, HRV and PWB. For a more strict analysis, partial correlation analyses were conducted after controlling for the cofounding factors (Table 7).

**TABLE 6:** Correlation coefficients of the associations of sex, age and social status with PA, HRV, and PWB.

	PA					HRV					Psychological Well‐Being (PWB)						
	Total PA	Sedentary time	Walking activity	Moderate PA	High PA	RMSSD (nu)	SDNN (nu)	NN50 (nu)	pNN50 (nu)	HF (nu)	**PWB** **total**	Purpose in life	Personal Growth	Positive relations	Autonomy	Self‐acceptance	Environmental mastery
Sex	−0.24	0.13	0.03	−0.38^**^	−0.31^*^	−0.33	−0.28	−0.38	−0.44^*^	−0.30	−0.08	−0.08	−0.14	−0.23	0.28	0.07	0.04
Age	−0.07	0.17	−0.07	0.05	−0.14	0.31	0.21	0.42^*^	0.43^*^	0.63^**^	0.05	0.01	0.05	0.29^*^	−0.16	−0.03	0.05
Social status	0.33^*^	−0.06	0.16	0.30^*^	0.24	0.14	0.12	0.22	0.22	0.42^*^	0.19	0.23	0.08	0.12	−0.11	0.05	0.09

*Note*: Significance level: ^*^p < 0.05, ^*^ ^*^ p < 0.01. The coefficients that were statistically significant are highlighted in bold.

**TABLE 7:** Partial correlation coefficients of the associations of psychophysiological factors with PA, mindfulness and PWB scores.

	PA					Mindfulness						Psychological well‐being (PWB)						
	**Total** **PA**	Sedentary time	Walking activity	Moderate PA	High PA	**FFMQ** **total**	Observation	Non‐reactivity	Non‐judging	Description	Acting with awareness	**PWB** **total**	Purpose in life	Personal Growth	Positive relations	Autonomy	Self‐acceptance	Environmental mastery
5‐HT (ng/mL)	0.00	0.24	0.23	−0.03	−0.13	−0.21	0.21	−0.37	−0.30	−0.15	0.00	−0.31	−0.24	0.04	−0.21	0.00	−0.42	−0.28
Oxytocin (ng/mL)	0.01	0.14	0.31	−0.24	0.01	0.05	−0.10	−0.03	0.28	−0.07	0.09	0.15	0.02	0.14	0.15	−0.07	0.11	0.27
Dopamine (pg/mL)	0.24	0.10	−0.07	−0.01	0.41	0.18	−0.01	0.19	0.00	0.16	0.22	0.15	0.07	0.20	0.05	−0.21	0.39	0.05
RMSSD (ms)	0.14	0.01	−0.23	0.47^*^	−0.05	−0.27	−0.33	−0.12	−0.34	−0.35	0.22	0.04	0.28	0.26	−0.33	0.11	−0.06	−0.11
SDNN (ms)	0.13	0.03	−0.17	0.42	−0.07	−0.25	−0.35	−0.11	−0.29	−0.33	0.24	0.10	0.30	0.29	−0.32	0.10	0.01	0.01
NN50	0.00	−0.24	−0.31	0.35	−0.09	0.04	0.09	−0.04	−0.28	0.05	0.28	0.33	0.50^*^	0.42	−0.23	0.26	0.18	0.19

*Note*: Significance level: ^*^ *p* < 0.05. The coefficients that were statistically significant are highlighted in bold.

Moderate‐intensity PA was found to be positively correlated with HF and the RMSSD (HRV), with correlation coefficients of *r* (17) = 0.47, *p* = 0.04 and *r* (17) = 0.47, *p* = 0.04, respectively. Additionally, NN50 (HRV) was positively correlated with the purpose in life dimension of the PWB scale, with a coefficient of *r* (17) = 0.50, *p* = 0.03. On the other hand, none of the HRV indices were significantly correlated with mindfulness (*p* > 0.05). Moreover, there were no correlations of any of the neurotransmitters/neuropeptides (5‐HT/OXT/DA levels) with PA, mindfulness, or PWB dimensions. All the correlations above remained significant after controlling for age, sex, and social status (student or working professional).

## 4 Discussion

The aim of the present study was to explore the potential psychophysiological factors associated with PA, mindfulness, and EWB. To investigate these relationships, we conducted Spearman's correlation analysis, followed by partial correlation analyses controlling for demographic factors (age, sex, and social status). Partial correlation analyses partly supported our hypothesis: the HRV indices (HF, RMSSD, and NN50) were positively correlated with both PA (moderate‐intensity PA) and EWB as measured by the purpose in life dimension of the PWB scale.

### 4.1 Relationships of PA With Psychophysiological Factors and PWB

These findings are in accordance with previous findings regarding the effectiveness of exercise on HRV and PWB scores. Kim et al. (2017) reported that 8 weeks of moderate‐intensity aerobic training (60% of heart rate reserve) significantly improved HRV (RMSSD, pNN50%), compared with the control condition in habitual smokers. Moreover, higher intensities (Kiviniemi et al. 2010) and a combination of moderate‐ and high‐intensity interventions (da Silva et al. 2019) seem to improve ANS function (Grässler et al. 2021). A greater HRV is associated with improved psychological functions, such as self‐regulatory capacity and adaptability or resilience, in the healthy population (Shaffer and Ginsberg 2017). Koval et al. (2013) reported that HRV was negatively related to instability of positive affect, indicating that individuals with lower parasympathetic tone are emotionally less stable. Additionally, higher levels of HRV are linked to improved executive functions such as attention and emotional processing by the prefrontal cortex (McCraty and Shaffer 2015). Given the above findings, exercise is effective for enhancing ANS function, and HRV is associated with better psychological functioning. As previously described, moderate‐ to high‐intensity PA is associated with higher PWB among younger populations (Lapa 2015; Ugwueze et al. 2021). In an intervention study, 8 weeks of a Zumba exercise program for young healthy women led to a meaningful increase in PWB (Delextrat et al. 2016). While the associations of HRV with PA and psychological functions have been observed independently, we suggest that PA could enhance PWB by influencing HRV modulation. Some findings support our suggestion. Recently, HRV‐guided training (HRVG), an individualized training method that adjusts training intensity on the basis of daily changes in the HRV, was shown to be effective in improving the resting HRV as measured by the RMSSD (Manresa‐Rocamora et al. 2021). da Silva et al. (2020) examined changes in mood state and recovery–stress perception in untrained women after HRVG and reported that engagement in HRVG decreased psychological symptoms (e.g., emotional stress, social stress, and mood disturbance) and increased self‐regulation. Moreover, HRVG had a greater effect on the reduction of several symptoms (social stress, lack of energy, tension, fatigue, and total mood disturbance) and led to a greater improvement in HRV (da Silva et al. 2019) than did predefined training. However, the causal relationship between HRV and PWB after an exercise intervention has not been examined; therefore, additional intervention studies are needed in the future to validate this causal relationship. To the best of our knowledge, lower levels or dysfunction of neurotransmitters have been observed in individuals with psychiatric disorders (e.g., depression), although it is unclear whether neurotransmitters/neuropeptides are related to positive aspects of mental health (e.g., positive affect and psychological function measured by PWB). Because altered neurotransmitter levels have been linked to mental and behavioral disorders (e.g., de Vries, van de Weijer, and Bartels 2022) and exercise is effective for improving mental disorders, we expected to observe positive correlations of 5‐HT, OXT, and DA levels with PWB and PA.

In contrast, we found inverse correlations of daily PA with 5‐HT and OXT levels as well as an inverse correlation between OXT levels and the autonomy dimension of the PWB scale, although these correlations were all weakened when the control variables were considered. Interestingly, however, several studies have reported a negative relationship between exercise and 5‐HT levels, similar to the findings of the present study. In two studies, different types of exercise (aerobic, anaerobic, and combined) interventions improved mental health (reduced depression, improved quality of life) and decreased peripheral 5‐HT levels (Wipfli et al. 2011; Pietta‐Dias et al. 2019). Similarly, a reduction in plasma 5‐HT levels was observed after several weeks of treatment with selective serotonin reuptake inhibitors (SSRIs), which are the most commonly prescribed antidepressants (Holck et al. 2019). Wipfli et al. (2011) suggested that exercise and SSRIs may have similar physiological effects, although the specific mechanism linking reductions in blood 5‐HT levels to changes in the central 5‐HT system remains unclear. On the other hand, Schroecksnadel et al. (2008) reported no relationship between tryptophan, a precursor of 5‐HT, and QoL or depression in HIV‐infected patients. Considering the limited number of prior studies and the lack of consistent outcomes, along with variations in sample characteristics across studies, it is imperative to examine the role of 5‐HT as a mediator of exercise‐induced improvements in mental health while considering a range of factors (e.g., sex, age, medical conditions).

OXT levels are closely associated with social behavior in both animals and humans, and intranasal administration of OXT has been shown to increase trust (Kosfeld et al. 2005). Additionally, OXT administration decreased anxiety and peripheral cortisol levels both before and after exposure to psychosocial stress (Heinrichs et al. 2003). Considering the above findings, we hypothesized that OXT levels are positively related to PWB (optimal human functioning), but no relationship was observed. Importantly, higher levels of peripheral OXT have been observed in individuals with social disorders such as autism spectrum disorder and social anxiety disorder (Hoge et al. 2008; Taylor, Saphire‐Bernstein, and Seeman 2010), although lower levels of anxiety and depression are linked to higher PWB (Liu, Shono, and Kitamura 2009). Moreover, Okazaki (1997) reported that individuals who value autonomy and prefer to make decisions on their own experience less social avoidance, social pain in social situations, and fear of social evaluation. In the present study, correlation analyses not adjusting for any other variables revealed that individuals with higher scores on the autonomy dimension of the PWB scale had a lower level of OXT. Given the aforementioned studies by Liu et al. and Okazaki et al., we speculate that individuals with a lower level of OXT and a higher level of PWB experience a lower level of anxiety. Regarding the relationship between exercise and peripheral OXT levels, some studies have reported that acute exercise (high‐intensity interval exercise) increases OXT levels (Tsukamoto et al. 2024), but no study has examined the effects of long‐term exercise on OXT levels in humans. Moreover, the relationship between increased OXT levels and mental states caused by exercise in humans has not yet been reported. Importantly, the relationships observed in the current study were diminished after adjusting for demographic factors. Takayanagi and Onaka (2022) concluded that it is undesirable to define OXT as a general biomarker of psychological states since the results regarding the relationship between OXT levels and psychological factors are complex. On the other hand, acute psychosocial stress (induced by the Trier Social Stress Test (TSST)) is suggested to increase both peripheral and central OXT levels, although these relationships have not been observed in resting conditions (Valstad et al. 2017). Therefore, assessing changes in OXT levels in response to acute stimuli could help reveal the role of the OXT system in psychological traits, including psychological function (EWB). While concerns regarding the influence of peripheral levels of neurotransmitters/neuropeptides on positive psychological states have been prevalent, this investigation is important because it involves exploratory research aimed at identifying psychophysiological markers associated with PA, mindfulness and PWB.

### 4.2 Relationships of Mindfulness With Psychophysiological Factors and PWB

Previous studies have reported that MBIs significantly increase tonic (resting‐state) HRV (e.g., RMSSD, HF, pNN50 values; Hunt et al. 2021; Joo et al. 2010; Park et al. 2018). On the other hand, the current study revealed that mindfulness (measured by FFMQ total scores) was positively related to scores on the PWB scale (*r*s = 0.60, *p* < 0.01), but one dimension of mindfulness was negatively correlated with HRV (HF, RMSSD, pNN50 values; see Figure 5 ). Because trait mindfulness was assessed via a self‐report survey, this unexpected relationship might have been affected by social desirability bias as previously reported (Brown and Ryan 2003). Individuals who are concerned about social desirability may have been inclined to overstate their level of mindfulness, potentially resulting in higher scores than their true level. Furthermore, individuals who employ a repressive coping style (repressors) typically deny the experience of distress despite observable physiological indices of stress (Weinberger, Schwartz, and Davidson 1979). Tamagawa et al. (2013) reported a correlation between mindfulness and repression. Given the aforementioned reports, within the context of this study, it is reasonable to speculate that suppressed cognitive processes could have been activated, potentially leading to lower HRV, despite the higher level of self‐reported mindfulness and PWB. It is imperative to examine the impact of mindfulness on overall well‐being in both psychological and physiological aspects while rigorously controlling for various psychosocial biases.

After controlling for demographic factors (age, sex, and social status), the relationship between mindfulness and HRV was weakened. These results suggest that demographic factors may affect the relationship between mindfulness and HRV. Therefore, future research should consider these variables as cofounding factors. Alternatively, the discrepancy between trait mindfulness and MBI impacts should be considered given the absence of a significant relationship between mindfulness and HRV in the present study. MBIs include elements such as focused awareness of the present moment, along with the observation of one's breathing. MBIs promote deep breathing and a reduction in the breathing rate, leading to improved HRV (Hunt et al. 2021). Furthermore, MBI‐induced increases in HRV have been suggested to mitigate psychological pain (Adler‐Neal et al. 2020). On the other hand, trait mindfulness reflects awareness of various life activities, such as “the sensation of one's body moving while walking,” but it does not encompass elements related to breathing. This suggests that it might not be mindful awareness per se but rather regulation of breathing that is relevant to HRV. Therefore, although a direct link between trait mindfulness and HRV was not apparent, studies should explore the importance of the respiratory control aspect, separate from mindful awareness in mindfulness practices, in enhancing HRV and PWB.

### 4.3 Future Perspectives

The novelty of this study lies in its examination of psychophysiological factors associated with PWB, along with PA and mindfulness. This pilot study uniquely contributes to the field by demonstrating that HRV is associated with both PA and PWB, thus supporting our hypothesis, whereas neurotransmitter levels did not show any such associations. As previously discussed, the relationships between neurotransmitters and EWB have not been well established, thus highlighting the importance and novelty of the current study. Nevertheless, further investigation is needed not only to understand the detailed processes underlying those relationships and their causality but also to explore the other potential psychophysiological factors related to both PA and PWB.

Cognitive (executive) function, especially inhibitory control (IC)—an integral component of self‐regulation—has been reported to have a significant relationship with HRV (Ottaviani et al. 2018), and both PA and MBIs have been shown to improve executive function and reduce perceived stress (de Bruin et al. 2016). In the present study, HRV emerged as the most prominent correlate of EWB. IC has been suggested to be a factor contributing to EWB (Toh and Yang 2022) and is regarded as a core element within self‐regulatory processes, including goal‐oriented actions and goal attainment (Hofmann et al. 2012; Mc Culloch et al. 2008). Therefore, it is possible that PWB is regulated through IC, on the basis of augmented HRV and PA.

Furthermore, a growing body of evidence suggests that EWB is associated with a reduction in the activation of the conserved transcriptional response to adversity (CTRA), which is characterized by the upregulation of genes involved in inflammation and the downregulation of genes involved in antiviral defenses (Cole et al. 2015; Fredrickson et al. 2013). Seeman et al. (2020) reported that the magnitude of individual decreases in CTRA gene expression was correlated with the magnitude of individual increases in EWB. Moreover, findings from breast cancer patients indicate that MBIs can significantly decrease the expression of the proinflammatory subcomponent of the CTRA, with a corresponding increase in EWB (Boyle et al. 2019). Recent studies have also reported a link between CTRA and HRV (Rahal et al. 2021; Sloan and Cole 2021). Because the PNS exerts a reciprocal inhibitory effect on inflammatory gene expression (Tracey 2002), it has been suggested that HRV affects the expression of genes such as CTRA, and there could be mutual associations among CTRA, HRV and EWB. However, these causal relationships have not yet been examined.

Additionally, other potential factors, such as cortisol and BDNF, may also be linked to well‐being (Farhud et al. 2014; Miao, Wang, and Sun 2020). In this study, we focused on HRV and neurotransmitters as candidate factors potentially related to positive aspects of well‐being. However, future research incorporating a wider range of well‐being‐related factors will allow for more comprehensive analyses and a deeper understanding of this field.

## 5 Summary

To the best of our knowledge, this study is the first to explore the relationships of PA and mindfulness with PWB, encompassing multiple psychophysiological factors (HRV and levels of 5‐HT, OXT, and DA). This study aimed to identify potential psychophysiological factors that may mediate the associations of PA and mindfulness with PWB. The primary outcomes of the current study were positive correlations among HRV, PA, and PWB even after controlling for relevant variables (age, sex, and social status). To establish causal relationships among PA, HRV, and PWB, future intervention studies utilizing HRVG are needed. Notably, certain inconsistencies observed in initial correlation analyses were resolved after the inclusion of relevant variables, leading to the conclusion that other psychophysiological factors (namely, 5‐HT, OXT, and DA levels) did not exhibit associations with PA, mindfulness, or PWB.

In light of the current study and recent research, it is imperative to conduct intervention studies to examine whether HRV might influence PA‐ or MBI‐induced PWB enhancement. In addition, other psychophysiological factors (e.g., neurotransmitters, executive function and CTRA) should be considered because of the current lack of substantiating evidence.

## 6 Limitations

While the present findings provide insight into the relationships between psychophysiological factors and PA and between mindfulness and EWB, it is important to acknowledge the limitations inherent in this study. First, the scope of this investigation was limited to exploring correlational associations between variables. To establish a more robust understanding of the causal relationships by which psychophysiological factors mediate the relationships of PA and mindfulness with PWB scores, future intervention studies are needed. Second, although we observed significant relationships among HRV, PA, and PWB scores, the sample size included in the analyses was relatively small (*n* = 26), primarily due to technical issues during ECG recording. This study did not include a priori power analysis in the methods section to determine an optimal sample size, which is typically recommended. Post hoc analysis via G^*^Power 3.1 revealed that the relationship between HRV and PWB was deemed sufficient (1 − *β* = 0.81), whereas insufficient statistical power (1 − *β* = 0.75) was detected in terms of the relationship between HRV and PA, as 1 − β was below the recommended threshold of 0.8 (Cohen 1988). Additionally, this study adopted an ECG sampling rate of 128 Hz, which is consistent with the minimum recommendation of 125 Hz for reliable HRV assessment in psychophysiological research (Laborde et al. 2017). The application of R‒R interval interpolation further ensured that the sampling rate was adequate for the accurate detection of HRV components in accordance with established guidelines (Laborde et al. 2017; Task Force 1996). However, more conservative recommendations suggest a sampling rate exceeding 200 Hz (Malik 1996) or between 500 and 1000 Hz. Future research with higher sampling rates could provide enhanced precision for analyzing subtle physiological variations while maintaining the accuracy of broader HRV assessments. To increase the generalizability of the findings, replication studies including larger and more diverse samples are recommended. Third, the assessment of psychophysiological factors was based on a single sample per participant. While efforts have been made to mitigate diurnal fluctuations by conducting all the procedures within a specific time frame (8:00 a.m. to 12:00 p.m.), the extent to which these outcomes accurately reflect individual characteristics remains uncertain. Both internal and external factors may have influenced the results, potentially obscuring intrinsic relationships. Therefore, future investigations should aim to capture the temporal dynamics of biological factors through repeated measurements to identify stable psychophysiological factors. Fourth, this study focused on only EWB rather than including other dimensions of psychological states, such as HWB and negative psychological states (e.g., anxiety and depression). Despite the distinction between EWB and HWB and psychological symptoms, these constructs are interconnected (Ryan and Deci 2001). Hence, to achieve a comprehensive understanding of mediating factors, especially EWB, future research that includes assessments of various psychological states, such as hedonic and negative psychological dimensions, is needed.

Finally, we targeted young adults because they are experiencing a period of psychological instability (Gruber et al. 2023), and an urgent solution is warranted. The effects of PA, such as aerobic exercise, on HRV (e.g., Grässler et al. 2021; Berger et al. 2019; Bouaziz et al. 2017; Villafaina et al. 2017) and PWB (Shams et al. 2021; Parra et al. 2020) have been repeatedly confirmed even among elderly and chronically ill patients (e.g., those with type 2 diabetes and psychiatric disorders), who are typically characterized by lower HRV. On the other hand, Sloan et al. (2017) failed to observe an association between HRV and PWB among middle‐older community‐dwelling samples (mean age = 55 years, SD = 16), and investigations between HRV and PWB have been limited in any population. Therefore, further research with diverse populations is warranted to clarify the relationships between psychophysiological indicators, PA, and PWB.

## Author Contributions

**Y. Murakami, H. Tsukamoto, K. Yamaura, and T. Hashimoto**: conceived and designed the research; **Y. Murakami**: performed the experiments; **Y. Murakami, and D. Goto**: analyzed the data; **Y. Murakami, H. Tsukamoto, D. Goto, K. Yamaura, and T. Hashimoto**: drafted the manuscript; **Y. Murakami, D. Goto, H. Tsukamoto, and T. Hashimoto**: edited and revised the manuscript; and all authors approved final version of the manuscript.

## Peer Review

The peer review history for this article is available at https://publons.com/publon/10.1002/brb3.70284.

## Data Availability

The data that support the findings of this study are openly available from the corresponding author, upon reasonable request.
